# Physical measures of physical functioning as prognostic factors in predicting outcomes for neck pain: Protocol for a prospective longitudinal cohort study

**DOI:** 10.1371/journal.pone.0354102

**Published:** 2026-07-17

**Authors:** Rabea Begum, Paul Parikh, David Walton, Alison Rushton

**Affiliations:** 1 School of Physical Therapy, Western University, London, Ontario, Canada; 2 CANSpine Research Group, Western University, London, Ontario, Canada; Universidade de Aveiro Escola Superior de Saude de Aveiro, PORTUGAL

## Abstract

**Introduction:**

Neck pain is a common musculoskeletal condition that contributes to significant global burden. Evidence on physical measures of functioning as prognostic factors for neck pain remains limited. The objective of this study is to investigate whether physical measures of physical functioning as prognostic factors predict outcomes for people with neck pain.

**Methods:**

This protocol for a prospective longitudinal cohort study will recruit approximately 156 adults with neck pain of any duration. Physical measures of physical functioning including impairment, performance, and activity measures in a real environment, will be assessed as prognostic factors. Outcomes at 12-month follow-up will include pain, disability, and overall changes, assessed using the Numeric Pain Rating Scale (NPRS), Neck Disability Index (NDI), and Global Rating of Change (GROC), respectively. Hierarchical regression analyses will be conducted separately for each physical measure and outcome. Physical measures will first be entered alone to examine the initial unadjusted (bivariate) association with the outcome, followed by adjustment for covariates (age, sex and baseline NPRS/NDI). Additional contribution will be evaluated using changes in explained variance ΔR^2^ and F-change statistics. Physical measures demonstrating additional contribution will subsequently be entered into the final penalized multivariable regression model using least absolute shrinkage and selection operator (LASSO) regression to reduce overfitting and identify the most relevant prognostic factors. Sensitivity analyses comparing penalized and non-penalized multivariable regression models will also be conducted to examine the consistency and stability of the findings. Prognostic effects will be reported as beta coefficients (β) or odds ratios (OR) with 95% confidence intervals (CIs). Each model will be internally validated using bootstrapping, with model performance evaluated using calibration and discrimination.

**Discussion:**

This study will provide new insights into how physical measures of physical functioning relate to outcomes for neck pain. The resulting prognostic models have the potential to strengthen clinical decision-making, support personalized care, and guide the development of targeted interventions for people with neck pain.

**Registration number:**

The protocol has prospectively registered on the Open Science Framework (OSF). The link is https://doi.org/10.17605/OSF.IO/HG6PJ.

## Introduction

Neck pain is a highly prevalent and debilitating musculoskeletal condition, currently ranked as the fourth leading cause of global years lived with disability [1]. Neck pain is best characterized as multifactorial and episodic in nature, often with an unclear etiology [2]. More than one-third of people with neck pain will develop chronic symptoms lasting more than six months [3,4] representing a serious health concern [5,6]. The socioeconomic burden of neck pain is therefore substantial, including long-term healthcare utilization and lost productivity [7]. People with neck pain demonstrate reduced physical functioning [8] which is one of the core domains recommended by clinical research guidelines [9,10].

Physical functioning provides evaluation of meaningful aspects of a person’s life, including the ability to perform household chores, walking, work, and self-care, as well as strength, endurance, and flexibility [9], categorized within the domains of the International Classification of Functioning, Disability, and Health (ICF) [11]. Physical functioning can be assessed using patient-reported outcome measures (PROMs, self-perceived function) or physical measures (observed function) [12,13]. PROMs are useful for providing perceived physical functioning from the patient’s perspective [14] but are insufficient to measure the multiple domains of physical functioning [15,16]. Physical measures can be classified as impairment-based measures (e.g., limited range of motion measured by a cervical range of motion, CROM device), performance-based measures (e.g., endurance of deep neck flexors measured using the Craniocervical Flexion Test, CCFT) or activity measures in a real environment (e.g., physical activity measured by accelerometry) [9,11–13]. Knowledge of the prognostic value of these measures will enable clinicians to better assess prognosis, individualize management, and potentially improve outcomes [17].

Our recent systematic review [18] synthesized existing evidence for physical measures of physical functioning for predicting outcomes in neck pain. Using the Grading of Recommendations Assessment, Development and Evaluation (GRADE) approach, the overall level of evidence was low to very low. Low level evidence supports that cervical movement velocity in the frontal plane was a potential predictor of short-term disability, while low to very low level evidence indicates that others impairment- and performance-based measures were not predictors of pain, disability or overall changes in the short-term (< 3-month) and medium-term (3–12-month) follow up for people with neck pain. For measures including cervical active range of motion (AROM), joint position error (JPE), and endurance of the neck flexors, the evidence was conflicting or inconclusive, underscoring the need for high-quality prospective research. The review also revealed key gaps: most studies focused mainly on ROM as impairment and endurance as performance measures, with none examined activity measures in real environments. Studies were disproportionately focused on acute whiplash populations, limiting generalizability, and few adjusted adequately for confounders.

This study seeks to address evidence gaps by investigating the predictive ability of physical measures of physical functioning for neck pain. The objective of this study is to investigate whether physical measures of physical functioning as prognostic factors predict outcomes for people with neck pain.

## Methods

### Study design

This prospective longitudinal inception cohort observational study will be conducted following the recommendations of the Prognosis Research Strategy framework (PROGRESS) [19]. Study design and procedures are informed by the Quality In Prognosis Studies (QUIPS) tool to address potential sources of bias in prognostic factor research [20]. Reporting will follow the Transparent Reporting of a multivariable prediction model for Individual Prognosis Or Diagnosis (TRIPOD) statement [21] (S1 Table). Data will be obtained from the wider COMPrehensive 360° Assessment of Spinal pain Study (COMPASS). Ethical approval was obtained from Western University Research Ethics Board (application no. 122736). The study has been registered with the Open Science Framework (OSF) prior to the start of data collection.

### Study settings

Data will be collected at two primary locations in London Ontario, Canada: one publicly funded hospital-based clinic and one non-profit community rehabilitation clinic in London, Ontario, Canada. Study recruitment will commence in October 13, 2025 and is expected to continue until mid-June, 2027.

### Participants

Adults aged 18 years or older with a primary complaint of neck pain, with or without arm symptoms, of any duration, will be eligible for this study. Exclusion criteria will be: anyone unable or unwilling to provide informed consent, unable to communicate in English, or with medical conditions that could affect neck symptoms or function, including space-occupying lesion (e.g., tumors, cysts), serious neurological disorders (e.g., spinal cord injury, myelopathy), disc infection, inflammatory disorders (e.g., ankylosing spondylitis), fibromyalgia, metabolic bone diseases (e.g., osteoporosis), cervical spine dislocation or fracture, and pain of non-neuromusculoskeletal origin.

### Study recruitment

**Fig 1:**
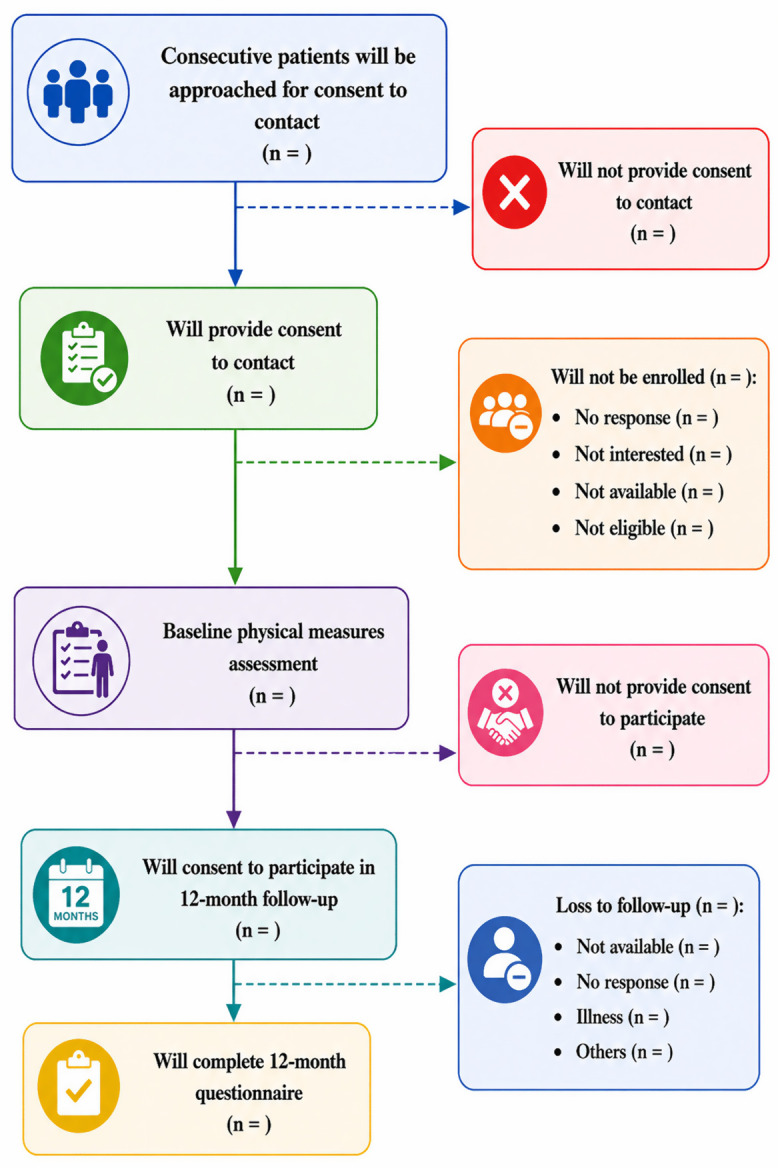
Study flow diagram. Overview of participants’ recruitment.

Consecutive participants will be approached to provide verbal consent to be contacted. Participants who will provide consent to be contacted will be first contacted through email with a letter of information. Participants will be contacted by phone call if they do not reply within 48 hours if they have any questions and to check their interest in taking part in the study. Participants who will provide consent, will be scheduled for an in-person data collection for baseline physical measures assessment at the CanMOVE Research Laboratory. After entering participant into study through EmPOWER database, participants will create their account and receive a unique ID number to link study participants with personal identifiers. Participants will fill out demographic and clinical variables surveys electronically (≈20 minutes), and will then undergo physical measures (≈30 minutes). Participants will complete 12-month outcome questionnaires through their EmPOWER account within 12 months (±1 month) after baseline assessment. Participants will fill out NPRS, NDI, and GROC at 12-month follow-up electronically (≈10 minutes). The study recruitment process is presented in Fig 1.

### Data management

All data will be entered into SPINA Spinal Rehabilitation Registry platform [22] through the EmPOWER Health Research Inc database, which has high standard of physical and virtual security for data management. Ethical approval for SPINA was obtained from Western University Research Ethics Board (application no. 122735) and Lawson Health Research Institute ReDA (application no. 13205). Only de-identified data will be used for analysis.

### Variables

Demographic variables including age, sex, height and weight to calculate body mass index (BMI), and clinical variables, such as types of symptoms (pain, numbness or pins and needles), and pain duration, will be recorded to describe the sample characteristics for descriptive reporting.

#### Physical measures of physical functioning as prognostic factors.

Prognostic factors (PFs) are selected based on current evidence synthesis regarding physical measures of physical functioning in predicting outcomes for neck pain [18]. The PFs are also chosen for their feasibility of measurement, as recommended in clinical practice guideline for neck pain [23], and their common use in clinical practice [24,25].

**Impairment-based measures:** In this study, cervical AROM, JPE, and handgrip strength will be assessed as impairment-based measures as they evaluate physical limitations or dysfunctions at the body structure or function level, in line with World Health Organization (WHO) International Classification of Functioning, Disability and Health (ICF) [11].

***Cervical AROM.*** Cervical AROM will be measured using the cervical range of motion (CROM) device (Performance Attainment Associates, Minnesota, USA) [26–28], which incorporates 3 embedded inclinometers to measure three-dimensional ROM with a single device attached to the participant’s head. The CROM device has demonstrated good–excellent intrarater reliability for measuring cervical AROM including all movements in individuals with neck pain (ICC_3,1_ = 0.87 to 0.94) and without neck pain (ICC_3,1_ = 0.88 to 0.96), [29], with good concurrent validity compared with the Fastrak (Polhemus, Colchester, Vermont, USA) system (r = 0.98 for extension and rotations, 0.93 for flexion [30]. Participants will be seated in a chair with feet flat on the floor and back supported. A strap will be used to gently restrict shoulders and trunk movement, isolating movements to the neck [30]. The CROM device will be placed on the participant’s head, and they will be instructed to move their head as far as possible comfortably in 4 directions: flexion, extension, right and left rotation [30]. Two trials will be performed for each movement direction, and the mean value of the two trials will be used for analysis. While some previous studies [31–34] have combined flexion and extension into a single sagittal plane motion value, in this study they will be analyzed separately as PFs, given their distinct biomechanical properties (i.e., involvement of the upper vs. lower cervical segments, respectively) [35], and their potential to reflect direction-specific functional impairments in individuals with neck pain [35,36], in accordance with previous studies [33,37–41]. For cervical rotation, left and right rotation will be combined to calculate total cervical rotation, which will be analyzed as a single predictor. This approach is considered appropriate because the study includes participants with both unilateral and bilateral neck pain, where direction-specific limitations may vary depending on the dominant or affected side. Combining left and right rotation therefore provides a more stable estimate of cervical AROM in rotation, consistent with previous studies [32,38,42–44].

***Cervical Joint Position Error (JPE).*** Cervical JPE will be assessed using the CROM device (Performance Attainment Associates, Minnesota, USA) [26–28]. JPE for rotation measured with the CROM device shows good to excellent intra-rater (ICC_3,1_ = 0.78–0.92) and inter-rater (ICC_2,1_ = 0.75–0.88) reliability in people with neck pain, with convergent validity supported between symptomatic and healthy individuals [45,46]. Rotation is selected as the most frequently reported movement for JPE in the literature for neck pain [46,47]. It has also shown greater deficits in JPE during left and right rotation rather than flexion and extension in neck pain population, suggesting that rotation is more sensitive for detecting cervical proprioceptive impairment [35,48,49]. For testing, the CROM device will be placed on the participant’s head. Participants will move their neck to the mid-point of their available rotational range and hold for 3 seconds to memorize this target position. After returning to the starting position, they will reproduce the target position as accurately as possible without feedback, holding for 3 seconds while the ROM will be recorded. The error between target and reproduced position (in degrees) will be recorded. Two trials will be performed for left and right rotation, and mean values will be calculated. Left and right rotation will be combined to calculate total cervical rotation for analysis as a single predictor to reduce variability from side-to-side differences and provide a stable estimate of overall cervical proprioception. This approach was considered appropriate because the study includes participants with both unilateral and bilateral neck pain, where side-specific measures may vary depending on the dominant or affected side. Combining both directions therefore provides a more consistent estimate of cervical JPE, and is also consistent with previous methods used in proprioception research for neck pain [50].

***Handgrip strength.*** Handgrip strength is a widely used physical measure of physical function and a useful indicator of general health status, and functional decline across various health conditions [51]. The handgrip strength for dominant hand will be measured by using a reliable and valid JTECH handgrip dynamometer (JTECH Medical, Midvale, Utah, USA) [52], in accordance with standardized protocol [53]. This device demonstrates good to excellent intra-rater reliability (ICC_2,1_ = 0.78–0.9) and inter-rater reliability (ICC_2,1_ = 0.76–0.95), with substantial concurrent validity for measuring handgrip strength in healthy adults [54]. For testing, the participant will be seated with feet flat, shoulder adducted, elbow flexed to 90°, forearm unsupported in neutral, writs positioned between 0–30° extension and 0–15° ulnar deviation. Participants will be instructed to squeeze as hard as they can. They will also receive encouragement while performing the test (e.g., “harder, harder, harder”) to facilitate them achieve maximum voluntary contraction. Two trials will be performed instead of three trials, to reduce to burden of assessment, with a 1 min rest interval. The handgrip strength, in kg, will be recorded and the mean value of 2 trials will be used for analysis, as this has previously been shown to be a more accurate reflection of true strength [55,56].

**Performance-based measure:** Performance-based measure assesses how well an individual perform specific tasks or actions in a standardized settings (e.g., hospital or clinic) [13]. Endurance of the deep neck flexors using the CCFT will be considered a performance-based measure as it requires participants to actively demonstrate graded activation and sustained control of the cervical flexor muscles in a standardized environment. This endurance test measures the activity level functioning (code: d220 in ICF coreset for musculoskeletal condition) defined by ICF.

***Endurance of deep neck flexor muscles.*** Endurance of the deep neck flexor muscles will be assessed using the CCFT with a pressure biofeedback unit (Stabilizer, Chattanooga Group, Hixson, TN, USA), with following standardized protocol as previously described [57]. The CCFT demonstrates good intra-rater reliability (ICC _3,1_ = 0.85–0.87) and inter-rater reliability (ICC_2,1_ = 0.78–0.81) in people with neck pain. Its construct validity is also supported by its ability to distinguish symptomatic from healthy individuals and by its correlation with deep cervical flexor thickness (r = 0.65–0.74) [58,59]. The test consists of two phases. In the activation phase, participants will gently nod to increase pressure in 2 mmHg at each level (22, 24, 26, 28, and 30 mmHg), holding each for 10 seconds while correct activation without superficial muscle substitution will be confirmed through observation or palpation. In the performance (endurance) phase, at the highest pressure achieved without substitution, participants will perform repeated 10-second holds with short rests between trials until they can no longer maintain the target pressure for 10 seconds without any compensatory movement or muscle activation. The highest pressure successfully held for 10 seconds will be recorded as the endurance measure.

**Activity measures in a natural environment:** ActiGraph activity monitor has been used in recent clinical research [60–63]. Physical activity and sleep will be objectively measured using the ActiGraph GT9X Link, a research-grade accelerometer [64–67]. Participants will wear the watch on their non-dominant wrist for 7 consecutive days and nights, ensuring comfort and compliance while maintaining accuracy and validity [68]. Only 5 continuous days with ≥10 hours (600 minutes) per day of wear time will be considered as valid measurement [69]. Participants will receive written instructions and a prepaid envelope for device return after the 7-day period. Data will be processed using the manufacturer’s validated algorithms, with mean values across valid days will be used for analysis. It is recommended to include physical activity measurements including both weekend and weekdays [70] as Gretebeck and Montoye [71] found no day-to-day difference when only weekdays were compared. Single-day ICCs for sleep can also be as low as 0.38 for weekdays and 0.27 for weekends; wearing the device for 7 days and nights achieves ICC of 0.7 for both [72], supporting include of both weekdays and weekend data.

***Physical activity.*** Physical activity will be objectively measured using ActiGraph GT9X Link accelerometer (ActiGraph LLC, Pensacola, FL, USA), which shows strong criterion validity for estimating physical activity [73,74] and good test–retest reliability in healthy adults (ICC _2,1_ = 0.806, 95% CI = 0.669–0.898) [74] for healthy adults. Physical activity level will be classified according to the Freedson Adult [75] cut-off points, a validated method developed for ActiGraph accelerometers. These cut-offs define sedentary activity as <100 counts per minute (cpm), light activity as 100–1,951 cpm, moderate activity as 1,952–5,724 cpm, vigorous activity as 5,725–9,498 cpm, and very vigorous activity as ≥9,499 cpm. Time in each category will be calculated in minutes per day, with moderate, vigorous, and very vigorous activity combined into a single moderate to vigorous physical activity (MVPA) category, following WHO physical activity guideline [76], consistent with previous literature [77–79]. These categories examined their independent associations with health outcomes in musculoskeletal pain and recovery [79]; however, the prognostic value of physical activity for neck pain does not exist due to insufficient evidence [18].

***Sleep.*** The sleep quantity and sleep efficiency will be objectively measured using ActiGraph GT9X Link accelerometer (ActiGraph LLC, Pensacola, FL, USA). The ActiGraph demonstrates sufficient concurrent validity and moderate test–retest reliability in healthy adults (ICC_2,1_ = 0.45 to 0.50) for sleep efficiency and ICC _2,1_ = 0.41 to 0.44 for total sleep time [16] for healthy adults [80]. Data will be processed with the manufacturer’s validated Cole–Kripke algorithm for adults, which has been validated against polysomnography among healthy adults [81]. Sleep quantity, expressed as TST (minutes/day) indicates how much sleep an individual obtains, and sleep quality as sleep efficiency (percentage of time in bed spent asleep), reflects how well an individual has slept Sleep quality is an important measure of sleep health [82], and provides additional information beyond sleep quantity [83], supporting its inclusion as a separate factor in prognostic modeling.

### Outcomes and outcome measures

Outcomes including pain, disability, and overall changes, will be measured at 12 months using patient-reported outcome measures (PROMs): NPRS, NDI, and GROC, respectively. PROMs that have previously demonstrated adequate validity and reliability in adults with neck pain [84,85].These instruments are widely recommended in neck pain research [84,86], and implementing an electronic PROMs survey is relatively easy and well accepted by patients [87,88].

#### Pain.

Pain intensity will be measured using the 11-point NPRS [89], which demonstrates moderate test–retest reliability for neck pain (ICC_2,1_ = 0.67; 95% CI: 0.27–0.84) and acceptable construct validity [90]. Participants will be asked to rate their average pain over the past week on a scale from 0 (no pain) to 10 scale (worst pain imaginable). Continuous NPRS scores will be used in the analyses to preserve information and support adequately powered linear regression modelling.

#### Disability.

Disability will be measured by using the NDI [91]. The NDI consists of 10 items covering daily activities including personal care, lifting, reading, work, driving, sleeping and recreation. Each item has five ordinal response options from 0 (no disability) to 5 (complete disability), producing a maximum total score of 50, which can be expressed as a percentage (0% to 100%). The NDI has demonstrated excellent test–retest reliability (ICC_2,1_ = 0.88; 95% CI: 0.63–0.95) and acceptable construct validity for mechanical neck pain population [90]. Continuous NDI scores will be used in the analyses to preserve information and support adequately powered linear regression modelling.

#### Overall changes.

Overall changes will be measured using the GROC [92], a 15-point ordinal scale, which has excellent test-retest reliability (ICC_2,1_ = 0.84; 95% CI: 0.65–0.94) for spinal population [93] and acceptable construct validity for people with neck disorders [94]. Participants will be asked to rate their neck condition at 7 levels of improvement (positive scores: “A tiny bit better” to “A very great deal better”), no change (0 score), or 7 levels of worsening (negative scores: “A tiny bit worse” to “A very great deal worse”). Previous studies [92,95] reported meaningful improvement ranges from 2 to 5 points of GROC scale for spinal pain population. In this study, participants reporting an improvement of ≥2 points (positive scores) will be classified as improved, while those reporting no change or worsening (negative scores) will be classified as not improved.

The study variables measured at baseline and 12-month follow-up are presented in Table 1.

**Table 1:** Summary of study variables.

Variables	Baseline assessment	12-month follow-up
Age in years	✓	
Sex (male/female)	✓	
BMI	✓	
Duration of symptoms	✓	
Pain (yes/no)	✓	
Numbness (yes/no)	✓	
Pins and needles (yes/no)	✓	
Pain, NPRS score (0–10 scale)	✓	✓
Disability, NDI score	✓	✓
Overall changes, GROC		✓
**Physical measures**		
*Impairment-based measures*		
AROM: flexion, extension and rotation (CROM device)	✓	
JPE: rotation (CROM device)	✓	
Handgrip strength (dynamometer)	✓	
*Performance-based measures*		
Endurance of deep neck flexor (CCFT test)	✓	
*Activity measures in a real environment*		
Sleep: sleep quantity and quality (ActiGraph)	✓	
Physical activity: sedentary, light, and moderate to vigorous physical activity (ActiGraph)	✓	

### Covariates

Age and sex will be included as covariates in the hierarchical regression model to account for factors that may influence both prognostic factors and outcomes, consistent with previous prognostic studies for neck pain [96–100]. Age and sex are associated with physical measures of physical functioning [101–104] and neck pain outcomes [105,106]. Increasing age is associated with reduced physical functioning (e.g., reduced strength), and higher pain and disability [107–109], while females demonstrate lower physical functioning (e.g., reduced strength), and higher pain intensity and disability [110–112]. Baseline pain intensity (NPRS) and disability (NDI) will also be included to examine whether physical measures provide additional prognostic information beyond baseline pain intensity and disability as individuals with higher baseline pain intensity and disability may demonstrate poorer physical functioning and different outcomes over time. Adjustment for these variables will help account for their shared variance with both physical functioning measures and outcomes after partialling out the variance explained by age, sex, and baseline outcome measures (NPRS, NDI).

**Fig 2:**
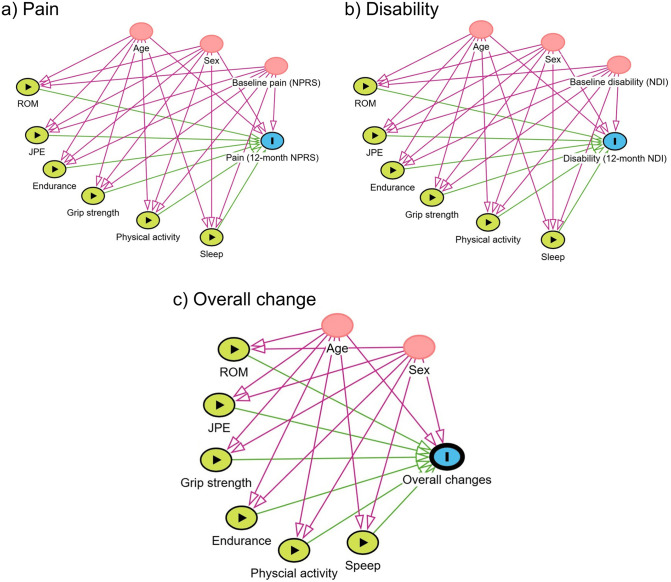
Directed acyclic graph: Green nodes are exposures, blue nodes are outcomes, and pink nodes are covariates.

The Directed Acyclic Graph [113] is constructed using DAGitty tool [114] (Fig 2) as a conceptual framework to illustrate the relationship between prognostic factors as exposures and outcomes (pain, disability and overall change).

### Bias

Several strategies will be implemented to minimize potential sources of bias throughout the study. Selection bias will be minimized by recruiting participants using predefined eligibility criteria and standardized recruitment procedures across study sites. Measurement bias will be reduced through the use of standardized outcome measures and physical assessment procedures conducted by trained assessors. Assessors will follow standardized testing protocols to reduce measurement bias and improve consistency of data collection.

### Sample size

This study will examine whether 15 variables (Table 1) predict 12-month pain, disability, and overall change outcomes using multivariable linear and logistic regression analyses. To support prognostic model development and reduce the risk of overfitting, sample size considerations were informed by both conventional and contemporary recommendations for prognostic modelling. Conventional recommendations of at least 10 participants per predictor variable are considered to support adequately powered linear regression analyses, resulting in an estimated minimum sample size of 150 participants for 15 candidate parameters. Because the shrinkage method using LASSO penalized regression produces models with fewer retained predictors [115], the final prognostic models are expected to include fewer predictors than the initial candidate set. In addition, applying the contemporary Riley et al. framework [116] for continuous outcomes, using 15 variables, a shrinkage factor of 0.90, and an anticipated model *R*^2^ of 0.63 [41] produced a comparable estimated sample size of approximately 125 participants for adequately powered linear regression. After accounting for an anticipated 20% attrition rate, the recruitment target was set at 156 participants.

For logistic regression analyses using the LASSO penalized regression to examine overall change (GROC) as a binary outcome, a minimum of 5 events per predictor variable was considered sufficient for model development [115]. Based on 15 variables, at least 75 events were required. Using an anticipated event rate of 62% [117], the estimated minimum sample size is 121 participants. After adjusting for an anticipated 20% attrition rate, the required sample size increases to 151 participants.

### Statistical analyses

All statistical analyses will be performed by R software (Rstudio, version 4.5.0) [118]. A *p* value of <0.05 will be considered statistically significant for all analyses, except variable selection. Descriptive statistics will summarize sample characteristic: continuous variables as mean with standard deviation, and categorical variables as numbers with percentages. Loss to follow-up will be reported descriptively, including the number of participants lost to follow-up and reasons for non-participation at the 12-month follow-up where available. Baseline characteristics of completers and non-completers will also be compared using independent t-tests or Mann–Whitney U tests for continuous variables and Chi-squared (χ^2^) or Fisher’s exact tests for categorical variables to assess potential attrition bias.

Prior to model development, data screening and missing data patterns will be examined. Descriptive statistics will be used to summarize participant characteristics, prognostic factors, and outcomes. Separate regression models will be developed for the continuous outcomes of pain (NPRS) and disability, and the binary outcome of overall change (GROC), dichotomized as improved versus not improved.

Model assumptions will be assessed before regression analyses, including linearity, normality of residuals, homoscedasticity, independence of errors, multicollinearity, and linearity of continuous predictors with the logit where appropriate. Multicollinearity among candidate prognostic factors will be assessed using variance inflation factors (VIF), with VIF values >5 considered indicative of potential multicollinearity.

#### Variable selection and model development.

Hierarchical regression analyses will be conducted separately for each physical measure and each outcome. In Step 1, the physical measure will be entered alone to examine the initial unadjusted (bivariate) association with the outcome. In Step 2, age and sex will be added to examine the adjusted multivariable association between the physical measure and the outcome after adjusting for age and sex. InStep 3, baseline NPRS/NDI (depending on the outcome) will be added to determine whether the physical measure provides additional contribution beyond baseline NPRS/NDI. Steps 2 and 3 will examine whether the physical measure contributes to the outcome after partialling out the variance explained by age, sex, and baseline outcome measures. Additional contribution will be evaluated using changes in explained variance (Δ*R*^2^) [119] and F-change statistics [120].

Following this, candidate predictors demonstrating significant F-change statistics (*p* < 0.10) [120] and meaningful ΔR^2^ (≥2.5%) in the hierarchical regression analyses will be entered together with confounders and covariates into a final penalized multivariable regression model using least absolute shrinkage and selection operator (LASSO) regression [121]. LASSO regression applies coefficient shrinkage coefficients toward zero to reduce overfitting and improve model stability when multiple predictors are included in the same model. This approach is consistent with current recommendations for prognostic model development to reduce overfitting [122]. The optimal penalty parameter will be selected using the one-standard-error criterion, lamda.1se (λ.1SE) from cross-validation to promote a more parsimonious and stable model [123]. Separate penalized multivariable linear regression models will be developed for the NPRS and NDI outcomes, while a penalized multivariable logistic regression model will be developed for the GROC outcome.

Prognostic effects will be reported as beta coefficients (β) or odds ratios (OR) with 95% confidence intervals (CIs) and corresponding p values. β values equal to 0 indicate no change in the outcome, values >0 indicate increased outcome scores, and values <0 indicate decreased outcome scores. OR values equal to 1 indicate no association with the outcome, values >1 indicate increased odds of the outcome, and values <1 indicate decreased odds of the outcome.

**Fig 3:**
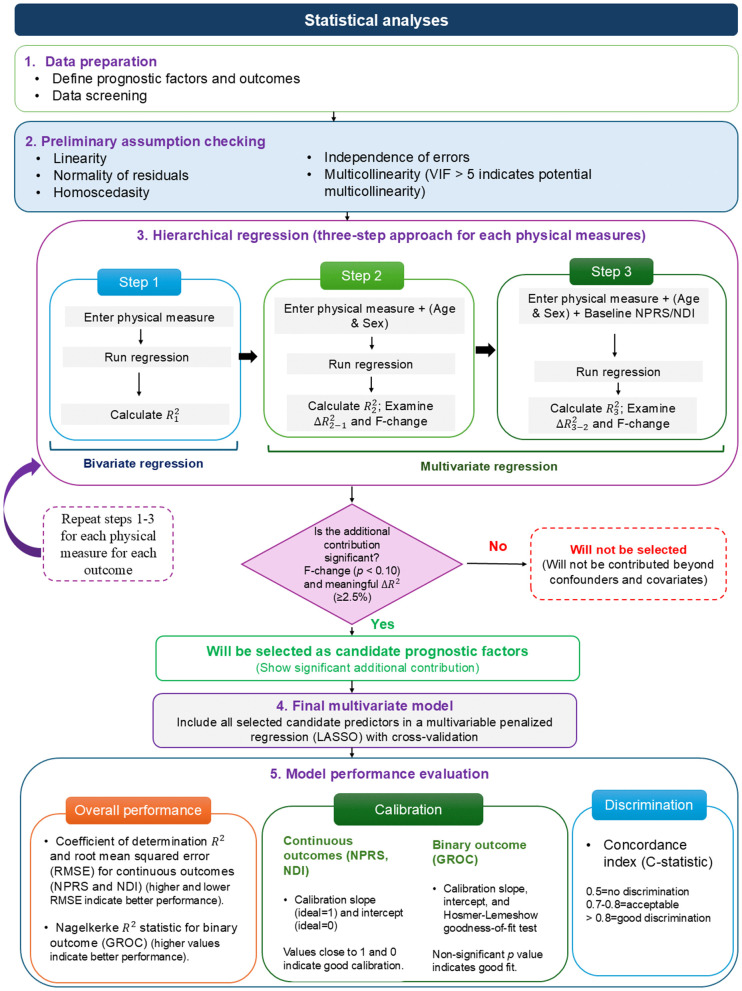
Statistical analyses workflow. The figure illustrates the model development process.

Sensitivity analyses comparing penalized and non-penalized multivariable regression models will be conducted to examine the consistency and stability of the findings. The model development workflow is presented in Fig 3.

#### Model performance.

Predictive performance of the prognostic models [124] will be evaluated by examining overall performance, calibration, and discrimination. For the continuous NPRS and NDI outcomes, overall performance will be assessed using the coefficient of determination (*R*^2^) and root mean squared error (RMSE), where higher *R*^2^ values and lower RMSE values indicate better model performance. For the binary GROC outcome, overall performance will be assessed using the Nagelkerke *R*^2^ statistic [125], where higher values indicate better model performance.

Calibration reflects the agreement between predicted and observed outcomes. For the continuous NPRS and NDI outcomes, calibration will be evaluated using the calibration slope and intercept, with values close to 1 and 0 indicating good calibration, respectively. For the binary GROC outcome, calibration will be assessed using calibration slope, calibration intercept, and the Hosmer– Lemeshow statistic goodness-of-fit test [126], where a non-significant p value indicates good model fit.

Discrimination reflects the model’s ability to distinguish between participants with different outcomes. For the binary GROC outcome, discrimination will be assessed using the concordance index (C-statistic) to evaluate the model’s ability to distinguish between participants who do and do not experience improvement, where values of 0.5 indicate no discrimination, values between 0.7 and 0.8 indicate acceptable discrimination, and values >0.8 indicate good discrimination. To account for model optimism and assess stability, internal validation will be conducted using bootstrap resampling with 1,000 iterations [127].

### Handling of missing data

Missing values will be imputed using the multiple imputation by chained equations (MICE) method [128]. Missing predictor and 12-month follow-up outcome data will be imputed under the assumption that data are missing at random (MAR). Imputation will be supported by Little’s test of data missing completely at random (p < 0.05) [129]. Sensitivity analyses [130] will be conducted by comparing estimates from complete-case analyses and imputed datasets to assess how missing data handling impacts model performance and results. Complete case analysis excludes participants with missing data, which can result in biased parameter estimates and poor predictive performance if the missingness is related to the outcome [131]. The comparison evaluates differences in discrimination and calibration, with results often showing imputed dataset provides more accurate and less biased predictions [132]. Comparison of the effect estimated (e.g., β, OR) between complete-case and imputed dataset will help us to understand differences in the identified predictors.

### Subgroup or interaction analyses

No subgroup analyses are planned a priori. However, hierarchical regression analyses will examine whether physical measures provide additional prognostic contribution beyond age, sex.

### Patient and public involvement

This study is developed in close collaboration with the Spinal Pain Patient Partner Advisory Group (PPAG), School of Physical Therapy, Western University. Patient partners were engaged through regular meetings with the CANSpine clinical research group, School of Physical Therapy, Western University, an established forum for discussion and co-design. Through these meetings, patient partners provided critical input in two key areas. First, they supported the inclusion of activity measures in a real environment as potential PFs, such as sleep quality and physical activity, highlighting their relevance to the lived experience of neck pain. Second, they emphasized the importance of using a validated research tool for activity measure (e.g., accelerometer), rather than relying on smartwatches. These insights directly influenced the selection of PFs in the study. The PPAG will continue to be engaged through ongoing CANSpine meetings, including participation in interpretating findings and contributing to public dissemination of results.

## Discussion

This prospective longitudinal observational cohort study aims to investigate whether physical measures of physical functioning including AROM, JPE and handgrip strength as impairment-based measures, endurance test for deep neck flexor as performance-based measure, physical activity and sleep as activity measures in a real environment, predict 12-month pain, disability, and overall changes for people with neck pain. By focusing on physical functioning, the study will provide a more robust understanding of how physical measures contribute to future outcomes for neck pain population. The analysis will be guided by contemporary methodological recommendation for prognostic modeling. The prognostic model will be developed using hierarchical regression analyses, followed by penalized regression using LASSO for final model development. Traditional events-per-variable [115] and contemporary Riley’s framework [116] were used to estimate sample size, and model performance will be using discrimination and calibration metrics, enhancing the methodological rigor of the final model. The selected prognostic factors are included based on our current evidence syntheses [18], relevance to clinical practice, and patient partner feedback.

The findings of this study will add to the literature by addressing the limited evidence identified in our systematic review [18], clarifying the prognostic role of novel physical measures and outcomes in people with neck pain. The knowledge gain through this study may inform clinical decision-making by identifying which physical measures can help predict outcomes for people with neck pain. If the model demonstrates adequate performance and calibration, the findings may support prognostic assessment in people with neck pain using physical measures of physical functioning. This could also support early patient identification, personalized treatment planning, goal setting, and the use of simple clinical tools for the management of neck pain. Additionally, the integration of objective and measurable physical measures into prognostic modeling reflects a shift toward more functionally meaningful data-driven care in musculoskeletal rehabilitation. Future work will be needed to externally validate the model and assess its impact on clinical outcomes, healthcare resource utilization, and patient-centered care.

The study incorporates several methodological features anticipated to strengthen its internal validity and applicability, including its prospective nature, inclusion of participants with different types and duration of neck pain, and inclusion of a coherent candidate set of PFs informed by our recent systematic review. Additional features include the use of comprehensive physical measures of physical functioning, including impairment, performance, and activity measures in a real environment, rigorous prognostic model development using multivariate linear and logistic regression analyses with model performance and internal validation procedures, blinded data analysis and transparent reporting aligned with contemporary best-practice recommendations for prognostic research. Anticipated limitations relate to external validity. First, the lack of external validation within the current study will limit the immediate generalizability of the prognostic model; future research will be required to validate the model in independent cohorts. Second, the observational design of the study limits the ability to infer causal relationships between physical measures and outcomes.

## Conclusion

This study will provide new evidence on the prognostic value of physical measures of physical functioning as PFs for long-term neck pain outcomes. By integrating impairment, performance-based, and activity measure in a real environment within a rigorous prognostic modelling and validation framework, the results may have the potential to inform personalized rehabilitation strategies and shape future research on prognostic tools in neck pain.

## Supporting information

S1 Table TRIPOD checklist. Checklist of items reported according to the TRIPOD reporting guideline.
(DOCX)
