# Supplementary material for: Physical measures of physical functioning as prognostic factors in predicting outcomes for neck pain: Protocol for a prospective longitudinal cohort study
Source: PLoS One. 2026 Jul 17;21(7):e0354102. doi: 10.1371/journal.pone.0354102 (PMC13379007; doi:10.1371/journal.pone.0354102)
Supplement: Attachment — Submitted filename: Response to Editor and Reviewer.docx [file pone.0354102.s003.docx]

**Feedback from Editor**

Journal Requirements:

When submitting your revision, we need you to address these additional requirements.

1. Please ensure that your manuscript meets PLOS ONE's style requirements, including those for file naming. The PLOS ONE style templates can be found at

<https://journals.plos.org/plosone/s/file?id=wjVg/PLOSOne_formatting_sample_main_body.pdf> and

<https://journals.plos.org/plosone/s/file?id=ba62/PLOSOne_formatting_sample_title_authors_affiliations.pdf>

**Action(s) performed:** We have reviewed the manuscript and ensured that it complies with PLOS ONE’s style and formatting requirements, including file naming, figure legends, table formatting, supplementary information, and font sizes according to the level of headings. We added the Supporting Information section, including the TRIPOD checklist, at the end of the manuscript in accordance with PLOS ONE recommendations (lines 1126 to 1128). In addition, all figures were removed from the manuscript file and uploaded separately according to PLOS ONE submission requirements.

2. Thank you for stating the following in the Acknowledgments Section of your manuscript: 
“No specific funding is received for this study”

We note that you have provided funding information that is not currently declared in your Funding Statement. However, funding information should not appear in the Acknowledgments section or other areas of your manuscript. We will only publish funding information present in the Funding Statement section of the online submission form.

Please remove any funding-related text from the manuscript and let us know how you would like to update your Funding Statement. Currently, your Funding Statement reads as follows: 
“The author(s) received no specific funding for this work.”
Please include your amended statements within your cover letter; we will change the online submission form on your behalf.

**Action(s) performed:** We have removed the funding-related text (“No specific funding is received for this study”) from the Acknowledgements section of the manuscript, in accordance with journal guidelines. We confirm that the correct Funding Statement is: “The author(s) received no specific funding for this work.”

3. If the reviewer comments include a recommendation to cite specific previously published works, please review and evaluate these publications to determine whether they are relevant and should be cited. There is no requirement to cite these works unless the editor has indicated otherwise.

**Answer:** No reviewer-recommended citations were provided; therefore, no changes were required.

**Additional Editor Comments:**

This manuscript presents a protocol for a prospective longitudinal cohort study investigating physical measures of physical functioning as prognostic factors for neck pain outcomes. The topic is relevant and timely, and the study is well aligned with established methodological frameworks (TRIPOD, PROGRESS, QUIPS). The inclusion of impairment-based, performance-based, and real-world activity measures is a notable strength. However, important methodological concerns—particularly regarding statistical modeling strategy, confounder handling, and outcome dichotomization—need to be addressed to ensure the robustness and validity of the proposed analyses.

**Major Comments**
1. The proposed use of univariate screening followed by stepwise regression is a major methodological concern, as this approach is prone to overfitting and model instability. More robust alternatives (e.g., penalized regression or theory-driven variable selection) should be considered.

**Action(s) performed:** Thank you for this comment. In response, we revised the statistical analysis plan and removed the use of univariate screening as a variable selection approach due to concerns regarding overfitting and model instability.

Instead, hierarchical regression analyses will be conducted to examine the additional contribution of each physical measure beyond covariates: age, sex, baseline pain intensity and disability. Additional contribution will be evaluated using changes in explained variance (ΔR²) and F-change statistics. Candidate predictors demonstrating meaningful contribution in the hierarchical analyses will then be entered together with these variables into a final penalized multivariable regression model using Least Absolute Shrinkage and Selection Operator (LASSO) regression. The hierarchical analyses will examine whether physical measures contribute to the outcome after partialling out the variance explained by age, sex, and baseline outcome measures. This approach is adopted to improve model stability, reduce overfitting, and align the analysis with more robust contemporary prognostic modelling recommendations.

Sensitivity analyses comparing penalized and non-penalized models have also been added to assess the robustness of the findings. We also added a statistical workflow diagram to improve clarity and better illustrate the overall analytical process (Fig 3, line 555).These revisions are incorporated throughout the Statistical Analysis section of the manuscript (lines 507 to 554).

2. The sample size justification based on the traditional events-per-variable rule is outdated and may not adequately ensure model stability.

**Action(s) performed:** Thank you for this important comment. In response, we revised the sample size justification by incorporating both conventional (events-per-variable) recommendations and the contemporary sample size framework proposed by Riley et al. (2019). Specifically, this framework considers the anticipated model R² and applies a shrinkage factor (e.g., 0.90) to minimize overfitting and improve the reliability and stability of prognostic models. In addition, because LASSO penalized regression is planned to reduce overfitting and retain fewer predictors in the final model, this was also considered in the revised sample size justification for both continuous and binary outcome analyses. The manuscript has been revised accordingly (lines 456 to 492).

Riley RD, Snell KI, Ensor J, Burke DL, Harrell Jr FE, Moons KG, Collins GS. Minimum sample size for developing a multivariable prediction model: Part I–Continuous outcomes. Statistics in medicine. 2019 Mar 30;38(7):1262-75.

3. The handling of confounders appears limited (age and sex only), which may result in residual confounding in a multifactorial condition such as neck pain.

**Action(s) performed:** Thank you for this important comment. We agree that neck pain is a multifactorial condition. However, this study is not designed to estimate a causal effect but rather as a prognostic study, where the primary goal is to predict outcomes. Therefore, to avoid causal terminology, age and sex are now described as covariates rather than confounders (Dyer, 2025).

Dyer BP. The distinction between causal, predictive, and descriptive research–there is still room for improvement. Journal of Clinical Epidemiology. 2025 Sep 1:111960.

Physical measures are included as the primary predictors of interest, while age and sex are considered as covariates based on prior literature to estimate the unique variance explained by each physical measure.

In addition, baseline pain intensity and disability are included as covariates to examine whether physical measures provide additional prognostic information beyond baseline pain intensity and disability. Adjustment for these variables will help account for their shared variance with both physical functioning measures and outcomes after partialling out the variance explained by age, sex, and baseline outcome measures (NPRS, NDI). Overall change (GROC) was not included as a baseline covariate because it is a follow-up outcome measure that reflects perceived change over time rather than baseline measure. All variables assessed at baseline and 12-month follow-up are now clearly presented in Table 1 of the revised manuscript (line 409).

4. The dichotomization of continuous outcomes (e.g., NPRS, NDI) leads to loss of information and reduced statistical power; this approach should be better justified or reconsidered.

**Action(s) performed:** Thank you for this important comment. In response, NPRS and NDI are now considered as continuous outcomes to preserve all available information and improve statistical power, consistent with PROGRESS recommendations and prognostic modelling guidance. This approach preserves the full variability of the NPRS and NDI scores and improves the efficiency of the analyses, using linear regression model. The manuscript has been revised accordingly (lines, 373 to 375; and 382 to 384). However, GROC was retained as a categorical outcome because it is an ordinal scale reflecting perceived overall change over time.

5. Some conceptual and wording issues (e.g., redundancy in study objectives) reduce clarity and should be revised.

**Action(s) performed:** We have revised the study objective to improve clarity and reduce redundancy (line 128).

**Minor Comments**
Improve clarity and conciseness of writing, avoiding repetition of key terms.

**Action(s) performed:** Thank you for this suggestion. We revised the manuscript to improve clarity and conciseness by streamlining sentences, reducing redundancy, and minimizing repetition of key terms throughout the text.

We also shortened some sections of the Introduction and Discussion (lines before 92, 100, 142, 723; tracked changes visible in the revised manuscript). However, some methodological details were intentionally retained because previous evidence syntheses identified unclear definitions of prognostic factor measurements and outcomes as an important source of high risk of bias in prognostic research. Therefore, we aimed to provide sufficient detail to improve transparency and reproducibility of the study protocol.

Correct minor grammatical and typographical errors throughout the manuscript.

**Action(s) performed:** Thank you for this comment. We have carefully reviewed the manuscript and corrected minor grammatical and typographical errors (e.g., corrected a typographical error identified in Fig 1. Study flow diagram) throughout.

Clarify methodological decisions such as combining left/right measures.

**Action(s) performed:** We thank the reviewer for this important comment. We have now clarified the rationale for combining left and right cervical rotation measures in the manuscript.

Left and right rotation will be combined because the study includes participants with both unilateral and bilateral neck pain, combining left and right rotation into a total cervical rotation measure was considered more appropriate to reduce variability related to pain side dominance and provide a more stable estimate of cervical AROM and JPE in rotation. This approach is also consistent with previous research in neck pain. We have revise the manuscript for ROM (lines 251 to 254) and JPE (lines 271 to 276).

Improve readability of figures (e.g., DAG).

**Action(s) performed:** Thank you for this comment. We have improved the readability of the DAG figures by increasing the figure size and improving spacing (Fig 2).

Ensure the data availability statement complies with PLOS ONE policies.

**Action(s) performed:** Thank you for this comment. We have carefully checked that the Data Availability Statement complies with PLOS ONE requirements. The statement was removed from the Acknowledgements section and provided in the online submission system in accordance with PLOS ONE guidelines.

**Reviewer’s comments**

Reviewer #1: This is a really nice, tight protocol—methodologically it’s already very aligned with good prognostic research practice. Below is a STROBE based appraisal, adapted for the fact that this is a protocol (so all “Results” items are necessarily not applicable yet).The design clearly stated in title and abstract. The abstract succinctly covers background, objectives, design, sample size, prognostic factors, outcomes, main analyses, and registration. There is a strong, up to date rationale and evidence gap. The introduction clearly sets burden, conceptual framing, and evidence gap, including your own systematic review There are clear primary objective; the prognostic focus is explicit and aligned with prognostic model research. Key elements of the study design are presented early, explicitly and are acceptable. The setting location and recruitment period are clearly specified. The inclusion/exclusion and recruitment pathway are well described. Outcomes, prognostic factors, and confounders are clearly defined and justified, including ICF framing and psychometrics. For example: “Outcomes include pain (NPRS), disability (NDI), and overall change (GROC)… Age and sex will be included as potential confounding variables…” Measurement protocols and their psychometrics are detailed for each variable (CROM, JPE, handgrip, CCFT, ActiGraph, PROMs). This area is very strong. Thes ample size justification is clear and uses the events per variable (EPV) criterion for logistic regression. Handling of continuous variables and dichotomisation of outcomes is described, including MCIDs and MVPA categories. Univariate screening, stepwise multivariable logistic regression, adjustment for age and sex, and EPV considerations are all described. Clear plan for MICE method and sensitivity analyses. Sensitivity analyses comparing complete case vs imputed models are planned. STROBE expects reporting of realised numbers, descriptive data, outcome events, and effect estimates. These will be addressed in the eventual results paper (you already have a flow diagram template and clear outcome definitions, which will make this straightforward). You plan internal validation (bootstrapping) and sensitivity analyses, which fits here: “Internal validation will be conducted using bootstrap resampling with 1,000 iterations.” Discussion, limitations, interpretation, generalisability are anticipated. You summarise anticipated contribution rather than actual results, which is appropriate for a protocol. Anticipated limitations are clearly discussed: “Anticipated limitations relate to external validity. First, the lack of external validation… Second, the choice of logistic regression over linear modelling…” Interpretation is cautious and grounded in the evidence gap and methodological choices. You explicitly discuss external validity and the need for external validation in independent cohorts. Funding and competing interests are transparent.

**Minor recommendations of areas that could be improved follow:**
1. Several biases addressed but could be slightly more explicit. If you wanted to be even more STROBE explicit, you could add a short subsection titled “Bias” summarizing: selection bias, information bias (blinding of assessors if applicable), and strategies to minimize loss to follow up.

**Action(s) performed:** Thank you for this helpful suggestion. In response, a separate Bias section has been added to the Methods section (lines 448 to 454). This section now outlines strategies that will be implemented to minimize potential sources of bias, including selection and measurement bias.

2. No explicit subgroup or interaction analyses are planned. For STROBE completeness, you could add a sentence stating that no interaction terms are planned a priori (or specify any if you decide to).

**Action(s) performed:** Thank you for highlighting this point. In response, we added a statement clarifying that no subgroup analyses are planned a priori (lines 626 to 628). However, the hierarchical regression analyses will examine whether physical measures provide additional prognostic contribution beyond age, sex, and baseline outcome measures.

3. Loss to follow up is implicitly handled via missing data strategy; you also pre specify that 19 participants are expected not to participate in 12 month follow up in the flow diagram. A brief explicit statement on how attrition will be described (numbers, reasons) would fully tick the STROBE box.

**Action(s) required:** Thank you for highlighting this point. The number ‘19’ shown in the previous flow diagram (Fig 1) was a typographical error and has now been removed from the revised figure. As this is a study protocol, participant follow-up data have not yet been collected. Loss to follow-up and attrition will be reported in the final study flow diagram, including the number of participants lost to follow-up and reasons for non-participation. We revised the entire study flow diagram (Fig 1) to improve clarity.

We also added that baseline characteristics of completers and non-completers will be compared to assess potential attrition bias (lines 496 to 502). In addition, the Handling of Missing Data section further describes the planned multiple imputation and sensitivity analyses to address missing predictors and outcome data related to loss to follow-up (lines 615, 616).

4. Very little is missing, but if you’d like it to read like a future STROBE perfect results paper:
- Add a short “Bias” subsection explicitly naming selection, information, and attrition bias and how each is mitigated.
- Add one sentence on subgroups/interactions (e.g., “No subgroup or interaction analyses are planned a priori” or specify any you might consider).
- Add an explicit statement on describing loss to follow up (numbers and reasons) in the planned reporting.

**Action(s) required:** Thank you for these thoughtful recommendations and for the positive feedback regarding the overall reporting quality of the manuscript. In response, we have incorporated the suggested revisions to further improve STROBE completeness. Specifically, we added a separate ‘Bias’ subsection describing strategies to minimize selection, measurement, and follow-up-related bias, included a statement clarifying that no subgroup or interaction analyses are planned a priori, and clarified that the numbers and reasons for non-participation in the 12-month follow-up will be described in the final study report. The manuscript has been revised accordingly.
