# Supplementary material for: Physical measures of physical functioning as prognostic factors in predicting outcomes for neck pain: Protocol for a prospective longitudinal cohort study
Source: PLoS One. 2026 Jul 17;21(7):e0354102. doi: 10.1371/journal.pone.0354102 (PMC13379007; doi:10.1371/journal.pone.0354102)
Supplement: Attachment — Submitted filename: Response to Editor and Reviewer_Revision2.docx [file pone.0354102.s004.docx]

Thank you for the opportunity to revise and resubmit our manuscript. We carefully reviewed all editorial comments and revised the manuscript accordingly. Specifically, we reviewed and updated the reference list to ensure accuracy, completeness, and consistency, and we assessed all figures using the PLOS New Automated Art Screening System (NAAS) to ensure compliance with PLOS ONE figure requirements.

Below, we provide a detailed point-by-point response to each editorial comment.

**Journal Requirements:**

If the reviewer comments include a recommendation to cite specific previously published works, please review and evaluate these publications to determine whether they are relevant and should be cited. There is no requirement to cite these works unless the editor has indicated otherwise.

**Response:** Thank you for this guidance. We reviewed all references included in the manuscript and confirmed that they were selected based on their relevance to the study objectives, methodology, and reporting framework. No references were added solely because they were recommended; all cited works were evaluated for relevance and appropriateness before inclusion.

Please review your reference list to ensure that it is complete and correct. If you have cited papers that have been retracted, please include the rationale for doing so in the manuscript text, or remove these references and replace them with relevant current references. Any changes to the reference list should be mentioned in the rebuttal letter that accompanies your revised manuscript. If you need to cite a retracted article, indicate the article’s retracted status in the References list and also include a citation and full reference for the retraction notice.

**Action(s) performed:** Thank you for this important recommendation. We carefully reviewed the entire reference list to verify the accuracy, completeness, and appropriateness of all cited works. We also assessed the references for potential retractions and found no evidence that any of the cited articles had been retracted.

As part of this review, several references were corrected and updated to improve completeness and accuracy. Specifically, references 6 and 8 were updated with volume and issue information; reference 11 with the appropriate URL; reference 14 with page information; reference 18 with volume, issue, and page information; references 19 and 22 with revised titles; reference 28 with page information; reference 71 with issue and page information; reference 77 with the appropriate URL; reference 89 with the publication year; reference 116 with page information; and reference 119 was updated. In addition, reference 63 was removed because we were unable to verify the publication. The redundant reference 81 was removed. References 13 and 57 were identified as duplicate citations of the same publication; therefore, reference 13 was removed and reference 57 was retained.

All corresponding changes have been incorporated into the revised manuscript and updated reference list.

**Additional Editor Comments :**

Dear Authors,
The manuscript is of interest; congratulations. I recommend that the authors follow the reviewer's recommendations to enhance the manuscript.


[Note: HTML markup is below. Please do not edit.]


[NOTE: If reviewer comments were submitted as an attachment file, they will be attached to this email and accessible via the submission site. Please log into your account, locate the manuscript record, and check for the action link "View Attachments". If this link does not appear, there are no attachment files.]

To ensure your figures meet our technical requirements, please review our figure guidelines: <https://journals.plos.org/plosone/s/figures>

You may also use PLOS’s free figure tool, NAAS, to help you prepare publication quality figures: <https://journals.plos.org/plosone/s/figures#loc-tools-for-figure-preparation>.

NAAS will assess whether your figures meet our technical requirements by comparing each figure against our figure specifications.

**Action(s) performed:** Thank you for your positive assessment of our manuscript. We carefully reviewed the editorial comments and revised the manuscript accordingly. In addition, all figures were reviewed using the PLOS New Automated Art Screening System (NAAS) to assess compliance with PLOS ONE figure requirements.

***Figure 1:*** Figure 1 was reviewed using the PLOS New Automated Art Screening System (NAAS). NAAS identified a mismatch in file extension and figure dimensions. To meet PLOS ONE figure specifications, the submitted file was converted to TIFF format and the figure dimensions were adjusted to a width of 3.4 inches and a height of 5.1 inches. Following these modifications, the figure was confirmed to meet the required specifications ("Image is adjusted to match the specification"). The standardized figure file generated by NAAS was uploaded on 11 June 2026 at 18:12.

***Figure 2:*** Figure 2 was reviewed using the PLOS New Automated Art Screening System (NAAS). NAAS adjusted the figure dimensions to a width of 3.7 inches and a height of 3.2 inches to comply with PLOS ONE figure specifications. Following these adjustments, the figure was confirmed to meet all technical requirements ("Image complies with specification"). The standardized figure file generated by NAAS was uploaded on 11 June 2026 at 17:32.

***Figure 3:*** Figure 3 was reviewed using the PLOS New Automated Art Screening System (NAAS). NAAS adjusted the figure dimensions to a width of 2.1 inches and a height of 2.8 inches to comply with PLOS ONE figure specifications. Following these adjustments, the figure was confirmed to meet all technical requirements ("Image complies with specification"). The standardized figure file generated by NAAS was uploaded on 11 June 2026 at 17:34.

All revised figure files generated through NAAS have been uploaded with the resubmission.
