# Supplementary material for: Physical measures of physical functioning as prognostic factors in predicting outcomes for neck pain: Protocol for a prospective longitudinal cohort study
Source: PLoS One. 2026 Jul 17;21(7):e0354102. doi: 10.1371/journal.pone.0354102 (PMC13379007; doi:10.1371/journal.pone.0354102)
Supplement: Peer review history

# Peer review: Decision Letter 0

**DOI:** 10.1371/journal.pone.0354102.r001

**Authors:** Mario Lopes

13 Apr 2026

PONE-D-25-53898

Physical measures of physical functioning as prognostic factors in predicting outcomes for neck pain: Protocol for a prospective longitudinal cohort study.

PLOS One

Dear Dr. Begum,

Thank you for submitting your manuscript to PLOS ONE. After careful consideration, we feel that it has merit but does not fully meet PLOS ONE’s publication criteria as it currently stands. Therefore, we invite you to submit a revised version of the manuscript that addresses the points raised during the review process.

The manuscript presents a well-structured and clinically relevant study protocol addressing an important gap in prognostic research for neck pain. The integration of multiple domains of physical functioning, including real-world activity measures, is a clear strength and enhances the potential impact of the study. However, several methodological aspects require clarification and improvement, particularly the statistical modeling approach, sample size justification, and handling of confounders. Addressing these issues will significantly strengthen the rigor and transparency of the protocol. Minor revisions to improve clarity, consistency, and presentation are also recommended. Please follow the recommendations point by point.

Please submit your revised manuscript by May 28 2026 11:59PM. If you will need more time than this to complete your revisions, please reply to this message or contact the journal office at plosone@plos.org. When you're ready to submit your revision, log on to https://www.editorialmanager.com/pone/ and select the 'Submissions Needing Revision' folder to locate your manuscript file.

Please include the following items when submitting your revised manuscript:

- A letter that responds to each point raised by the academic editor and reviewer(s). You should upload this letter as a separate file labeled 'Response to Reviewers'.
- A marked-up copy of your manuscript that highlights changes made to the original version. You should upload this as a separate file labeled 'Revised Manuscript with Track Changes'.
- An unmarked version of your revised paper without tracked changes. You should upload this as a separate file labeled 'Manuscript'.

If you would like to make changes to your financial disclosure, please include your updated statement in your cover letter. Guidelines for resubmitting your figure files are available below the reviewer comments at the end of this letter.

If applicable, we recommend that you deposit your laboratory protocols in protocols.io to enhance the reproducibility of your results. Protocols.io assigns your protocol its own identifier (DOI) so that it can be cited independently in the future. For instructions see: https://journals.plos.org/plosone/s/submission-guidelines#loc-laboratory-protocols. Additionally, PLOS ONE offers an option for publishing peer-reviewed Lab Protocol articles, which describe protocols hosted on protocols.io. Read more information on sharing protocols at https://plos.org/protocols?utm_medium=editorial-email&utm_source=authorletters&utm_campaign=protocols.

As the corresponding author, your ORCID iD is verified in the submission system and will appear in the published article. PLOS supports the use of ORCID, and we encourage all coauthors to register for an ORCID iD and use it as well. Please encourage your coauthors to verify their ORCID iD within the submission system before final acceptance, as unverified ORCID iDs will not appear in the published article. *Only*  the individual author can complete the verification step; PLOS staff *cannot*  verify ORCID iDs on behalf of authors.

We look forward to receiving your revised manuscript.

Kind regards,

Mario Lopes, Ph.D

Academic Editor

PLOS One

Journal Requirements:

When submitting your revision, we need you to address these additional requirements.

1. Please ensure that your manuscript meets PLOS ONE's style requirements, including those for file naming. The PLOS ONE style templates can be found at

https://journals.plos.org/plosone/s/file?id=wjVg/PLOSOne_formatting_sample_main_body.pdf and

https://journals.plos.org/plosone/s/file?id=ba62/PLOSOne_formatting_sample_title_authors_affiliations.pdf

2. Thank you for stating the following in the Acknowledgments Section of your manuscript:

“No specific funding is received for this study”

We note that you have provided funding information that is not currently declared in your Funding Statement. However, funding information should not appear in the Acknowledgments section or other areas of your manuscript. We will only publish funding information present in the Funding Statement section of the online submission form.

Please remove any funding-related text from the manuscript and let us know how you would like to update your Funding Statement. Currently, your Funding Statement reads as follows:

“The author(s) received no specific funding for this work.”

Please include your amended statements within your cover letter; we will change the online submission form on your behalf.

3. If the reviewer comments include a recommendation to cite specific previously published works, please review and evaluate these publications to determine whether they are relevant and should be cited. There is no requirement to cite these works unless the editor has indicated otherwise.

Additional Editor Comments:

This manuscript presents a protocol for a prospective longitudinal cohort study investigating physical measures of physical functioning as prognostic factors for neck pain outcomes. The topic is relevant and timely, and the study is well aligned with established methodological frameworks (TRIPOD, PROGRESS, QUIPS). The inclusion of impairment-based, performance-based, and real-world activity measures is a notable strength. However, important methodological concerns—particularly regarding statistical modeling strategy, confounder handling, and outcome dichotomization—need to be addressed to ensure the robustness and validity of the proposed analyses.

Major Comments

The proposed use of univariate screening followed by stepwise regression is a major methodological concern, as this approach is prone to overfitting and model instability. More robust alternatives (e.g., penalized regression or theory-driven variable selection) should be considered.

The sample size justification based on the traditional events-per-variable rule is outdated and may not adequately ensure model stability.

The handling of confounders appears limited (age and sex only), which may result in residual confounding in a multifactorial condition such as neck pain.

The dichotomization of continuous outcomes (e.g., NPRS, NDI) leads to loss of information and reduced statistical power; this approach should be better justified or reconsidered.

Some conceptual and wording issues (e.g., redundancy in study objectives) reduce clarity and should be revised.

Minor Comments

Improve clarity and conciseness of writing, avoiding repetition of key terms.

Correct minor grammatical and typographical errors throughout the manuscript.

Clarify methodological decisions such as combining left/right measures.

Improve readability of figures (e.g., DAG).

Ensure the data availability statement complies with PLOS ONE policies.

[Note: HTML markup is below. Please do not edit.]

Reviewers' comments:

Reviewer's Responses to Questions

**Comments to the Author**

1. Does the manuscript provide a valid rationale for the proposed study, with clearly identified and justified research questions?

The research question outlined is expected to address a valid academic problem or topic and contribute to the base of knowledge in the field.

Reviewer #1: Yes

**********

2. Is the protocol technically sound and planned in a manner that will lead to a meaningful outcome and allow testing the stated hypotheses?

The manuscript should describe the methods in sufficient detail to prevent undisclosed flexibility in the experimental procedure or analysis pipeline, including sufficient outcome-neutral conditions (e.g. necessary controls, absence of floor or ceiling effects) to test the proposed hypotheses and a statistical power analysis where applicable. As there may be aspects of the methodology and analysis which can only be refined once the work is undertaken, authors should outline potential assumptions and explicitly describe what aspects of the proposed analyses, if any, are exploratory.

Reviewer #1: Yes

**********

3. Is the methodology feasible and described in sufficient detail to allow the work to be replicable?

Descriptions of methods and materials in the protocol should be reported in sufficient detail for another researcher to reproduce all experiments and analyses. The protocol should describe the appropriate controls, sample size calculations, and replication needed to ensure that the data are robust and reproducible.

Reviewer #1: Yes

**********

4. Have the authors described where all data underlying the findings will be made available when the study is complete?

The PLOS Data policy requires authors to make all data underlying the findings described in their manuscript fully available without restriction, with rare exception, at the time of publication. The data should be provided as part of the manuscript or its supporting information, or deposited to a public repository. For example, in addition to summary statistics, the data points behind means, medians and variance measures should be available. If there are restrictions on publicly sharing data—e.g. participant privacy or use of data from a third party—those must be specified.

Reviewer #1: Yes

**********

5. Is the manuscript presented in an intelligible fashion and written in standard English?

PLOS ONE does not copyedit accepted manuscripts, so the language in submitted articles must be clear, correct, and unambiguous. Any typographical or grammatical errors should be corrected at revision, so please note any specific errors here.

Reviewer #1: Yes

**********

6. Review Comments to the Author

Please use the space provided to explain your answers to the questions above and, if applicable, provide comments about issues authors must address before this protocol can be accepted for publication. You may also include additional comments for the author, including concerns about research or publication ethics.

You may also provide optional suggestions and comments to authors that they might find helpful in planning their study.

(Please upload your review as an attachment if it exceeds 20,000 characters)

Reviewer #1: This is a really nice, tight protocol—methodologically it’s already very aligned with good prognostic research practice. Below is a STROBE based appraisal, adapted for the fact that this is a protocol (so all “Results” items are necessarily not applicable yet).The design clearly stated in title and abstract. The abstract succinctly covers background, objectives, design, sample size, prognostic factors, outcomes, main analyses, and registration. There is a strong, up to date rationale and evidence gap. The introduction clearly sets burden, conceptual framing, and evidence gap, including your own systematic review There are clear primary objective; the prognostic focus is explicit and aligned with prognostic model research. Key elements of the study design are presented early, explicitly and are acceptable. The setting location and recruitment period are clearly specified. The inclusion/exclusion and recruitment pathway are well described. Outcomes, prognostic factors, and confounders are clearly defined and justified, including ICF framing and psychometrics. For example: “Outcomes include pain (NPRS), disability (NDI), and overall change (GROC)… Age and sex will be included as potential confounding variables…” Measurement protocols and their psychometrics are detailed for each variable (CROM, JPE, handgrip, CCFT, ActiGraph, PROMs). This area is very strong. Thes ample size justification is clear and uses the events per variable (EPV) criterion for logistic regression. Handling of continuous variables and dichotomisation of outcomes is described, including MCIDs and MVPA categories. Univariate screening, stepwise multivariable logistic regression, adjustment for age and sex, and EPV considerations are all described. Clear plan for MICE method and sensitivity analyses. Sensitivity analyses comparing complete case vs imputed models are planned. STROBE expects reporting of realised numbers, descriptive data, outcome events, and effect estimates. These will be addressed in the eventual results paper (you already have a flow diagram template and clear outcome definitions, which will make this straightforward). You plan internal validation (bootstrapping) and sensitivity analyses, which fits here: “Internal validation will be conducted using bootstrap resampling with 1,000 iterations.” Discussion, limitations, interpretation, generalisability are anticipated. You summarise anticipated contribution rather than actual results, which is appropriate for a protocol. Anticipated limitations are clearly discussed: “Anticipated limitations relate to external validity. First, the lack of external validation… Second, the choice of logistic regression over linear modelling…” Interpretation is cautious and grounded in the evidence gap and methodological choices. You explicitly discuss external validity and the need for external validation in independent cohorts. Funding and competing interests are transparent.

Minor recommendations of areas that could be improved follow:

1. Several biases addressed but could be slightly more explicit. If you wanted to be even more STROBE explicit, you could add a short subsection titled “Bias” summarizing: selection bias, information bias (blinding of assessors if applicable), and strategies to minimise loss to follow up.

2. No explicit subgroup or interaction analyses are planned. For STROBE completeness, you could add a sentence stating that no interaction terms are planned a priori (or specify any if you decide to).

3. Loss to follow up is implicitly handled via missing data strategy; you also pre specify that 19 participants are expected not to participate in 12 month follow up in the flow diagram. A brief explicit statement on how attrition will be described (numbers, reasons) would fully tick the STROBE box.

4. Very little is missing, but if you’d like it to read like a future STROBE perfect results paper:

- Add a short “Bias” subsection explicitly naming selection, information, and attrition bias and how each is mitigated.

- Add one sentence on subgroups/interactions (e.g., “No subgroup or interaction analyses are planned a priori” or specify any you might consider).

- Add an explicit statement on describing loss to follow up (numbers and reasons) in the planned reporting.

**********

7. PLOS authors have the option to publish the peer review history of their article (what does this mean?). If published, this will include your full peer review and any attached files.

If you choose “no”, your identity will remain anonymous but your review may still be made public.

**Do you want your identity to be public for this peer review?**  For information about this choice, including consent withdrawal, please see our Privacy Policy.

Reviewer #1: No

**********

[NOTE: If reviewer comments were submitted as an attachment file, they will be attached to this email and accessible via the submission site. Please log into your account, locate the manuscript record, and check for the action link "View Attachments". If this link does not appear, there are no attachment files.]

To ensure your figures meet our technical requirements, please review our figure guidelines: https://journals.plos.org/plosone/s/figures

You may also use PLOS’s free figure tool, NAAS, to help you prepare publication quality figures: https://journals.plos.org/plosone/s/figures#loc-tools-for-figure-preparation.

NAAS will assess whether your figures meet our technical requirements by comparing each figure against our figure specifications.

# Author comment: Author response to Decision Letter 1

**DOI:** 10.1371/journal.pone.0354102.r002

25 May 2026

Feedback from Editor

Journal Requirements:

When submitting your revision, we need you to address these additional requirements.

1. Please ensure that your manuscript meets PLOS ONE's style requirements, including those for file naming. The PLOS ONE style templates can be found at

https://journals.plos.org/plosone/s/file?id=wjVg/PLOSOne_formatting_sample_main_body.pdf and

https://journals.plos.org/plosone/s/file?id=ba62/PLOSOne_formatting_sample_title_authors_affiliations.pdf

Action(s) performed: We have reviewed the manuscript and ensured that it complies with PLOS ONE’s style and formatting requirements, including file naming, figure legends, table formatting, supplementary information, and font sizes according to the level of headings. We added the Supporting Information section, including the TRIPOD checklist, at the end of the manuscript in accordance with PLOS ONE recommendations (lines 1126 to 1128). In addition, all figures were removed from the manuscript file and uploaded separately according to PLOS ONE submission requirements.

2. Thank you for stating the following in the Acknowledgments Section of your manuscript:

“No specific funding is received for this study”

We note that you have provided funding information that is not currently declared in your Funding Statement. However, funding information should not appear in the Acknowledgments section or other areas of your manuscript. We will only publish funding information present in the Funding Statement section of the online submission form.

Please remove any funding-related text from the manuscript and let us know how you would like to update your Funding Statement. Currently, your Funding Statement reads as follows:

“The author(s) received no specific funding for this work.”

Please include your amended statements within your cover letter; we will change the online submission form on your behalf.

Action(s) performed: We have removed the funding-related text (“No specific funding is received for this study”) from the Acknowledgements section of the manuscript, in accordance with journal guidelines. We confirm that the correct Funding Statement is: “The author(s) received no specific funding for this work.”

3. If the reviewer comments include a recommendation to cite specific previously published works, please review and evaluate these publications to determine whether they are relevant and should be cited. There is no requirement to cite these works unless the editor has indicated otherwise.

Answer: No reviewer-recommended citations were provided; therefore, no changes were required.

Additional Editor Comments:

This manuscript presents a protocol for a prospective longitudinal cohort study investigating physical measures of physical functioning as prognostic factors for neck pain outcomes. The topic is relevant and timely, and the study is well aligned with established methodological frameworks (TRIPOD, PROGRESS, QUIPS). The inclusion of impairment-based, performance-based, and real-world activity measures is a notable strength. However, important methodological concerns—particularly regarding statistical modeling strategy, confounder handling, and outcome dichotomization—need to be addressed to ensure the robustness and validity of the proposed analyses.

Major Comments

1. The proposed use of univariate screening followed by stepwise regression is a major methodological concern, as this approach is prone to overfitting and model instability. More robust alternatives (e.g., penalized regression or theory-driven variable selection) should be considered.

Action(s) performed: Thank you for this comment. In response, we revised the statistical analysis plan and removed the use of univariate screening as a variable selection approach due to concerns regarding overfitting and model instability.

Instead, hierarchical regression analyses will be conducted to examine the additional contribution of each physical measure beyond covariates: age, sex, baseline pain intensity and disability. Additional contribution will be evaluated using changes in explained variance (ΔR²) and F-change statistics. Candidate predictors demonstrating meaningful contribution in the hierarchical analyses will then be entered together with these variables into a final penalized multivariable regression model using Least Absolute Shrinkage and Selection Operator (LASSO) regression. The hierarchical analyses will examine whether physical measures contribute to the outcome after partialling out the variance explained by age, sex, and baseline outcome measures. This approach is adopted to improve model stability, reduce overfitting, and align the analysis with more robust contemporary prognostic modelling recommendations.

Sensitivity analyses comparing penalized and non-penalized models have also been added to assess the robustness of the findings. We also added a statistical workflow diagram to improve clarity and better illustrate the overall analytical process (Fig 3, line 555).These revisions are incorporated throughout the Statistical Analysis section of the manuscript (lines 507 to 554).

2. The sample size justification based on the traditional events-per-variable rule is outdated and may not adequately ensure model stability.

Action(s) performed: Thank you for this important comment. In response, we revised the sample size justification by incorporating both conventional (events-per-variable) recommendations and the contemporary sample size framework proposed by Riley et al. (2019). Specifically, this framework considers the anticipated model R² and applies a shrinkage factor (e.g., 0.90) to minimize overfitting and improve the reliability and stability of prognostic models. In addition, because LASSO penalized regression is planned to reduce overfitting and retain fewer predictors in the final model, this was also considered in the revised sample size justification for both continuous and binary outcome analyses. The manuscript has been revised accordingly (lines 456 to 492).

Riley RD, Snell KI, Ensor J, Burke DL, Harrell Jr FE, Moons KG, Collins GS. Minimum sample size for developing a multivariable prediction model: Part I–Continuous outcomes. Statistics in medicine. 2019 Mar 30;38(7):1262-75.

3. The handling of confounders appears limited (age and sex only), which may result in residual confounding in a multifactorial condition such as neck pain.

Action(s) performed: Thank you for this important comment. We agree that neck pain is a multifactorial condition. However, this study is not designed to estimate a causal effect but rather as a prognostic study, where the primary goal is to predict outcomes. Therefore, to avoid causal terminology, age and sex are now described as covariates rather than confounders (Dyer, 2025).

Dyer BP. The distinction between causal, predictive, and descriptive research–there is still room for improvement. Journal of Clinical Epidemiology. 2025 Sep 1:111960.

Physical measures are included as the primary predictors of interest, while age and sex are considered as covariates based on prior literature to estimate the unique variance explained by each physical measure.

In addition, baseline pain intensity and disability are included as covariates to examine whether physical measures provide additional prognostic information beyond baseline pain intensity and disability. Adjustment for these variables will help account for their shared variance with both physical functioning measures and outcomes after partialling out the variance explained by age, sex, and baseline outcome measures (NPRS, NDI). Overall change (GROC) was not included as a baseline covariate because it is a follow-up outcome measure that reflects perceived change over time rather than baseline measure. All variables assessed at baseline and 12-month follow-up are now clearly presented in Table 1 of the revised manuscript (line 409).

4. The dichotomization of continuous outcomes (e.g., NPRS, NDI) leads to loss of information and reduced statistical power; this approach should be better justified or reconsidered.

Action(s) performed: Thank you for this important comment. In response, NPRS and NDI are now considered as continuous outcomes to preserve all available information and improve statistical power, consistent with PROGRESS recommendations and prognostic modelling guidance. This approach preserves the full variability of the NPRS and NDI scores and improves the efficiency of the analyses, using linear regression model. The manuscript has been revised accordingly (lines, 373 to 375; and 382 to 384). However, GROC was retained as a categorical outcome because it is an ordinal scale reflecting perceived overall change over time.

5. Some conceptual and wording issues (e.g., redundancy in study objectives) reduce clarity and should be revised.

Action(s) performed: We have revised the study objective to improve clarity and reduce redundancy (line 128).

Minor Comments

Improve clarity and conciseness of writing, avoiding repetition of key terms.

Action(s) performed: Thank you for this suggestion. We revised the manuscript to improve clarity and conciseness by streamlining sentences, reducing redundancy, and minimizing repetition of key terms throughout the text.

We also shortened some sections of the Introduction and Discussion (lines before 92, 100, 142, 723; tracked changes visible in the revised manuscript). However, some methodological details were intentionally retained because previous evidence syntheses identified unclear definitions of prognostic factor measurements and outcomes as an important source of high risk of bias in prognostic research. Therefore, we aimed to provide sufficient detail to improve transparency and reproducibility of the study protocol.

Correct minor grammatical and typographical errors throughout the manuscript.

Action(s) performed: Thank you for this comment. We have carefully reviewed the manuscript and corrected minor grammatical and typographical errors (e.g., corrected a typographical error identified in Fig 1. Study flow diagram) throughout.

Clarify methodological decisions such as combining left/right measures.

Action(s) performed: We thank the reviewer for this important comment. We have now clarified the rationale for combining left and right cervical rotation measures in the manuscript.

Left and right rotation will be combined because the study includes participants with both unilateral and bilateral neck pain, combining left and right rotation into a total cervical rotation measure was considered more appropriate to reduce variability related to pain side dominance and provide a more stable estimate of cervical AROM and JPE in rotation. This approach is also consistent with previous research in neck pain. We have revise the manuscript for ROM (lines 251 to 254) and JPE (lines 271 to 276).

Improve readability of figures (e.g., DAG).

Action(s) performed: Thank you for this comment. We have improved the readability of the DAG figures by increasing the figure size and improving spacing (Fig 2).

Ensure the data availability statement complies with PLOS ONE policies.

Action(s) performed: Thank you for this comment. We have carefully checked that the Data Availability Statement complies with PLOS ONE requirements. The statement was removed from the Acknowledgements section and provided in the online submission system in accordance with PLOS ONE guidelines.

Reviewer’s comments

Reviewer #1: This is a really nice, tight protocol—methodologically it’s already very aligned with good prognostic research practice. Below is a STROBE based appraisal, adapted for the fact that this is a protocol (so all “Results” items are necessarily not applicable yet).The design clearly stated in title and abstract. The abstract succinctly covers background, objectives, design, sample size, prognostic factors, outcomes, main analyses, and registration. There is a strong, up to date rationale and evidence gap. The introduction clearly sets burden, conceptual framing, and evidence gap, including your own systematic review There are clear primary objective; the prognostic focus is explicit and aligned with prognostic model research. Key elements of the study design are presented early, explicitly and are acceptable. The setting location and recruitment period are clearly specified. The inclusion/exclusion and recruitment pathway are well described. Outcomes, prognostic factors, and confounders are clearly defined and justified, including ICF framing and psychometrics. For example: “Outcomes include pain (NPRS), disability (NDI), and overall change (GROC)… Age and sex will be included as potential confounding variables…” Measurement protocols and their psychometrics are detailed for each variable (CROM, JPE, handgrip, CCFT, ActiGraph, PROMs). This area is very strong. Thes ample size justification is clear and uses the events per variable (EPV) criterion for logistic regression. Handling of continuous variables and dichotomisation of outcomes is described, including MCIDs and MVPA categories. Univariate screening, stepwise multivariable logistic regression, adjustment for age and sex, and EPV considerations are all described. Clear plan for MICE method and sensitivity analyses. Sensitivity analyses comparing complete case vs imputed models are planned. STROBE expects reporting of realised numbers, descriptive data, outcome events, and effect estimates. These will be addressed in the eventual results paper (you already have a flow diagram template and clear outcome definitions, which will make this straightforward). You plan internal validation (bootstrapping) and sensitivity analyses, which fits here: “Internal validation will be conducted using bootstrap resampling with 1,000 iterations.” Discussion, limitations, interpretation, generalisability are anticipated. You summarise anticipated contribution rather than actual results, which is appropriate for a protocol. Anticipated limitations are clearly discussed: “Anticipated limitations relate to external validity. First, the lack of external validation… Second, the choice of logistic regression over linear modelling…” Interpretation is cautious and grounded in the evidence gap and methodological choices. You explicitly discuss external validity and the need for external validation in independent cohorts. Funding and competing interests are transparent.

Minor recommendations of areas that could be improved follow:

1. Several biases addressed but could be slightly more explicit. If you wanted to be even more STROBE explicit, you could add a short subsection titled “Bias” summarizing: selection bias, information bias (blinding of assessors if applicable), and strategies to minimize loss to follow up.

Action(s) performed: Thank you for this helpful suggestion. In response, a separate Bias section has been added to the Methods section (lines 448 to 454). This section now outlines strategies that will be implemented to minimize potential sources of bias, including selection and measurement bias.

2. No explicit subgroup or interaction analyses are planned. For STROBE completeness, you could add a sentence stating that no interaction terms are planned a priori (or specify any if you decide to).

Action(s) performed: Thank you for highlighting this point. In response, we added a statement clarifying that no subgroup analyses are planned a priori (lines 626 to 628). However, the hierarchical regression analyses will examine whether physical measures provide additional prognostic contribution beyond age, sex, and baseline outcome measures.

3. Loss to follow up is implicitly handled via missing data strategy; you also pre specify that 19 participants are expected not to participate in 12 month follow up in the flow diagram. A brief explicit statement on how attrition will be described (numbers, reasons) would fully tick the STROBE box.

Action(s) required: Thank you for highlighting this point. The number ‘19’ shown in the previous flow diagram (Fig 1) was a typographical error and has now been removed from the revised figure. As this is a study protocol, participant follow-up data have not yet been collected. Loss to follow-up and attrition will be reported in the final study flow diagram, i

Attachment Submitted filename: Response to Editor and Reviewer.docx

# Editor report: Decision Letter 1

**DOI:** 10.1371/journal.pone.0354102.r003

**Authors:** Mario Lopes

8 Jun 2026

PONE-D-25-53898R1

Physical measures of physical functioning as prognostic factors in predicting outcomes for neck pain: protocol for a prospective longitudinal cohort study.

PLOS One

Dear Dr. Begum,

Thank you for submitting your manuscript to PLOS ONE. After careful consideration, we feel that it has merit but does not fully meet PLOS ONE’s publication criteria as it currently stands. Therefore, we invite you to submit a revised version of the manuscript that addresses the points raised during the review process.

The authors have made an excellent effort, producing this manuscript. Please follow the reviewer recommendations.

Please submit your revised manuscript by Jul 23 2026 11:59PM. If you will need more time than this to complete your revisions, please reply to this message or contact the journal office at plosone@plos.org. When you're ready to submit your revision, log on to https://www.editorialmanager.com/pone/ and select the 'Submissions Needing Revision' folder to locate your manuscript file.

Please include the following items when submitting your revised manuscript:

- A letter that responds to each point raised by the academic editor and reviewer(s). You should upload this letter as a separate file labeled 'Response to Reviewers'.
- A marked-up copy of your manuscript that highlights changes made to the original version. You should upload this as a separate file labeled 'Revised Manuscript with Track Changes'.
- An unmarked version of your revised paper without tracked changes. You should upload this as a separate file labeled 'Manuscript'.

If you would like to make changes to your financial disclosure, please include your updated statement in your cover letter. Guidelines for resubmitting your figure files are available below the reviewer comments at the end of this letter.

If applicable, we recommend that you deposit your laboratory protocols in protocols.io to enhance the reproducibility of your results. Protocols.io assigns your protocol its own identifier (DOI) so that it can be cited independently in the future. For instructions see: https://journals.plos.org/plosone/s/submission-guidelines#loc-laboratory-protocols. Additionally, PLOS ONE offers an option for publishing peer-reviewed Lab Protocol articles, which describe protocols hosted on protocols.io. Read more information on sharing protocols at https://plos.org/protocols?utm_medium=editorial-email&utm_source=authorletters&utm_campaign=protocols.

As the corresponding author, your ORCID iD is verified in the submission system and will appear in the published article. PLOS supports the use of ORCID, and we encourage all coauthors to register for an ORCID iD and use it as well. Please encourage your coauthors to verify their ORCID iD within the submission system before final acceptance, as unverified ORCID iDs will not appear in the published article. *Only*  the individual author can complete the verification step; PLOS staff *cannot*  verify ORCID iDs on behalf of authors.

We look forward to receiving your revised manuscript.

Kind regards,

Mario Lopes, Ph.D

Academic Editor

PLOS One

Journal Requirements:

If the reviewer comments include a recommendation to cite specific previously published works, please review and evaluate these publications to determine whether they are relevant and should be cited. There is no requirement to cite these works unless the editor has indicated otherwise.

Please review your reference list to ensure that it is complete and correct. If you have cited papers that have been retracted, please include the rationale for doing so in the manuscript text, or remove these references and replace them with relevant current references. Any changes to the reference list should be mentioned in the rebuttal letter that accompanies your revised manuscript. If you need to cite a retracted article, indicate the article’s retracted status in the References list and also include a citation and full reference for the retraction notice.

Additional Editor Comments :

Dear Authors,

The manuscript is of interest; congratulations. I recommend that the authors follow the reviewer's recommendations to enhance the manuscript.

[Note: HTML markup is below. Please do not edit.]

[NOTE: If reviewer comments were submitted as an attachment file, they will be attached to this email and accessible via the submission site. Please log into your account, locate the manuscript record, and check for the action link "View Attachments". If this link does not appear, there are no attachment files.]

To ensure your figures meet our technical requirements, please review our figure guidelines: https://journals.plos.org/plosone/s/figures

You may also use PLOS’s free figure tool, NAAS, to help you prepare publication quality figures: https://journals.plos.org/plosone/s/figures#loc-tools-for-figure-preparation.

NAAS will assess whether your figures meet our technical requirements by comparing each figure against our figure specifications.

# Author comment: Author response to Decision Letter 2

**DOI:** 10.1371/journal.pone.0354102.r004

15 Jun 2026

Thank you for the opportunity to revise and resubmit our manuscript. We carefully reviewed all editorial comments and revised the manuscript accordingly. Specifically, we reviewed and updated the reference list to ensure accuracy, completeness, and consistency, and we assessed all figures using the PLOS New Automated Art Screening System (NAAS) to ensure compliance with PLOS ONE figure requirements.

Below, we provide a detailed point-by-point response to each editorial comment.

Journal Requirements:

If the reviewer comments include a recommendation to cite specific previously published works, please review and evaluate these publications to determine whether they are relevant and should be cited. There is no requirement to cite these works unless the editor has indicated otherwise.

Response: Thank you for this guidance. We reviewed all references included in the manuscript and confirmed that they were selected based on their relevance to the study objectives, methodology, and reporting framework. No references were added solely because they were recommended; all cited works were evaluated for relevance and appropriateness before inclusion.

Please review your reference list to ensure that it is complete and correct. If you have cited papers that have been retracted, please include the rationale for doing so in the manuscript text, or remove these references and replace them with relevant current references. Any changes to the reference list should be mentioned in the rebuttal letter that accompanies your revised manuscript. If you need to cite a retracted article, indicate the article’s retracted status in the References list and also include a citation and full reference for the retraction notice.

Action(s) performed: Thank you for this important recommendation. We carefully reviewed the entire reference list to verify the accuracy, completeness, and appropriateness of all cited works. We also assessed the references for potential retractions and found no evidence that any of the cited articles had been retracted.

As part of this review, several references were corrected and updated to improve completeness and accuracy. Specifically, references 6 and 8 were updated with volume and issue information; reference 11 with the appropriate URL; reference 14 with page information; reference 18 with volume, issue, and page information; references 19 and 22 with revised titles; reference 28 with page information; reference 71 with issue and page information; reference 77 with the appropriate URL; reference 89 with the publication year; reference 116 with page information; and reference 119 was updated. In addition, reference 63 was removed because we were unable to verify the publication. The redundant reference 81 was removed. References 13 and 57 were identified as duplicate citations of the same publication; therefore, reference 13 was removed and reference 57 was retained.

All corresponding changes have been incorporated into the revised manuscript and updated reference list.

Additional Editor Comments :

Dear Authors,

The manuscript is of interest; congratulations. I recommend that the authors follow the reviewer's recommendations to enhance the manuscript.

[Note: HTML markup is below. Please do not edit.]

[NOTE: If reviewer comments were submitted as an attachment file, they will be attached to this email and accessible via the submission site. Please log into your account, locate the manuscript record, and check for the action link "View Attachments". If this link does not appear, there are no attachment files.]

To ensure your figures meet our technical requirements, please review our figure guidelines: https://journals.plos.org/plosone/s/figures

You may also use PLOS’s free figure tool, NAAS, to help you prepare publication quality figures: https://journals.plos.org/plosone/s/figures#loc-tools-for-figure-preparation.

NAAS will assess whether your figures meet our technical requirements by comparing each figure against our figure specifications.

Action(s) performed: Thank you for your positive assessment of our manuscript. We carefully reviewed the editorial comments and revised the manuscript accordingly. In addition, all figures were reviewed using the PLOS New Automated Art Screening System (NAAS) to assess compliance with PLOS ONE figure requirements.

Figure 1: Figure 1 was reviewed using the PLOS New Automated Art Screening System (NAAS). NAAS identified a mismatch in file extension and figure dimensions. To meet PLOS ONE figure specifications, the submitted file was converted to TIFF format and the figure dimensions were adjusted to a width of 3.4 inches and a height of 5.1 inches. Following these modifications, the figure was confirmed to meet the required specifications ("Image is adjusted to match the specification"). The standardized figure file generated by NAAS was uploaded on 11 June 2026 at 18:12.

Figure 2: Figure 2 was reviewed using the PLOS New Automated Art Screening System (NAAS). NAAS adjusted the figure dimensions to a width of 3.7 inches and a height of 3.2 inches to comply with PLOS ONE figure specifications. Following these adjustments, the figure was confirmed to meet all technical requirements ("Image complies with specification"). The standardized figure file generated by NAAS was uploaded on 11 June 2026 at 17:32.

Figure 3: Figure 3 was reviewed using the PLOS New Automated Art Screening System (NAAS). NAAS adjusted the figure dimensions to a width of 2.1 inches and a height of 2.8 inches to comply with PLOS ONE figure specifications. Following these adjustments, the figure was confirmed to meet all technical requirements ("Image complies with specification"). The standardized figure file generated by NAAS was uploaded on 11 June 2026 at 17:34.

All revised figure files generated through NAAS have been uploaded with the resubmission.

Attachment Submitted filename: Response to Editor and Reviewer_Revision2.docx

# Editor report: Decision Letter 2

**DOI:** 10.1371/journal.pone.0354102.r005

**Authors:** Mario Lopes

2 Jul 2026

Physical measures of physical functioning as prognostic factors in predicting outcomes for neck pain: protocol for a prospective longitudinal cohort study

PONE-D-25-53898R2

Dear Dr. Begum,

We’re pleased to inform you that your manuscript has been judged scientifically suitable for publication and will be formally accepted for publication once it meets all outstanding technical requirements.

Within one week, you’ll receive an e-mail detailing the required amendments. When these have been addressed, you’ll receive a formal acceptance letter and your manuscript will be scheduled for publication.

An invoice will be generated when your article is formally accepted. Please note, if your institution has a publishing partnership with PLOS and your article meets the relevant criteria, all or part of your publication costs will be covered. Please make sure your user information is up-to-date by logging into Editorial Manager at Editorial Manager® and clicking the ‘Update My Information' link at the top of the page. For questions related to billing, please contact billing support.

If your institution or institutions have a press office, please notify them about your upcoming paper to help maximize its impact. If they’ll be preparing press materials, please inform our press team as soon as possible -- no later than 48 hours after receiving the formal acceptance. Your manuscript will remain under strict press embargo until 2 pm Eastern Time on the date of publication. For more information, please contact onepress@plos.org.

Kind regards,

Mario Lopes, Ph.D

Academic Editor

PLOS One

Additional Editor Comments (optional):

Dear author,

Thank you for taking into consideration the suggestions made. The manuscript has improved considerably.

Reviewers' comments:

# Editor report: Acceptance letter

**DOI:** 10.1371/journal.pone.0354102.r006

**Authors:** Mario Lopes

PONE-D-25-53898R2

PLOS One

Dear Dr. Begum,

I'm pleased to inform you that your manuscript has been deemed suitable for publication in PLOS One. Congratulations! Your manuscript is now being handed over to our production team.

At this stage, our production department will prepare your paper for publication. This includes ensuring the following:

* All references, tables, and figures are properly cited

* All relevant supporting information is included in the manuscript submission,

* There are no issues that prevent the paper from being properly typeset

You will receive further instructions from the production team, including instructions on how to review your proof when it is ready. Please keep in mind that we are working through a large volume of accepted articles, so please give us a few days to review your paper and let you know the next and final steps.

Lastly, if your institution or institutions have a press office, please let them know about your upcoming paper now to help maximize its impact. If they'll be preparing press materials, please inform our press team within the next 48 hours. Your manuscript will remain under strict press embargo until 2 pm Eastern Time on the date of publication. For more information, please contact onepress@plos.org.

You will receive an invoice from PLOS for your publication fee after your manuscript has reached the completed accept phase. If you receive an email requesting payment before acceptance or for any other service, this may be a phishing scheme. Learn how to identify phishing emails and protect your accounts at https://explore.plos.org/phishing.

If we can help with anything else, please email us at customercare@plos.org.

Thank you for submitting your work to PLOS ONE and supporting open access.

Kind regards,

PLOS ONE Editorial Office Staff

on behalf of

Prof. Mario Lopes

Academic Editor

PLOS One
